# Individual Differences in Premotor Brain Systems Underlie Behavioral Apathy

**DOI:** 10.1093/cercor/bhv247

**Published:** 2015-11-12

**Authors:** Valerie Bonnelle, Sanjay Manohar, Tim Behrens, Masud Husain

**Affiliations:** 1 Department of Experimental Psychology, University of Oxford, Oxford OX1 3UD, UK; 2 Institute of cognitive neuroscience, University College London, London WC1N 3AR, UK; 3 Department of Clinical Neurology, Centre for Functional Magnetic Resonance Imaging of the Brain,University of Oxford, John Radcliffe Hospital, Oxford OX3 9DU, UK; 4 Wellcome Trust Centre for Neuroimaging, University College London, London WC1N 3BG, UK; 5 Nuffield Department of Clinical Neurosciences, University of Oxford, Oxford OX3 9DU, UK

**Keywords:** action initiation, anterior cingulate cortex, cingulum bundle, motivation, supplementary motor area

## Abstract

Lack of physical engagement, productivity, and initiative—so-called “behavioral apathy”—is a common problem with significant impact, both personal and economic. Here, we investigate whether there might be a biological basis to such lack of motivation using a new effort and reward-based decision-making paradigm, combined with functional and diffusion-weighted imaging. We hypothesized that behavioral apathy in otherwise healthy people might be associated with differences in brain systems underlying either motivation to act (specifically in effort and reward-based decision-making) or in action processing (transformation of an intention into action). The results demonstrate that behavioral apathy is associated with increased effort sensitivity as well as greater recruitment of neural systems involved in action anticipation: supplementary motor area (SMA) and cingulate motor zones. In addition, decreased structural and functional connectivity between anterior cingulate cortex (ACC) and SMA were associated with increased behavioral apathy. These findings reveal that effort sensitivity and translation of intentions into actions might make a critical contribution to behavioral apathy. We propose a mechanism whereby inefficient communication between ACC and SMA might lead to increased physiological cost—and greater effort sensitivity—for action initiation in more apathetic people.

## Introduction

What makes some people motivated to act and pursue their goals, while others are seemingly apathetic, generating little enthusiasm for purposeful behavior? Why do some people work harder than others for the same rewards? These issues are important because lack of motivation has a significant societal and personal cost, affecting education and employment performance as well as civic engagement (Vansteenkiste et al. 2005). Despite a substantial body of research into the psychological processes underlying normal human motivation (Heckhausen and Heckhausen 2010), surprisingly little is known about neurobiological mechanisms that might account for apathy in otherwise healthy people.

Recent research on pathological apathy in patients with brain disorders has led to dissociation of apathy into several domains, for example: behavioral, cognitive, and emotional (Levy and Dubois 2006; Sockeel et al. 2006; Robert et al. 2009; Radakovic and Abrahams 2014). Here, we focus on “behavioral apathy”—lack of motivation to initiate behavior or respond to environmental stimuli. One approach to understanding the brain basis for this form of apathy is to consider the cognitive, motor, and neural mechanisms involved in deciding to engage in an effortful action. Studies of decision-making have suggested that there might be several underlying processes including: evaluation of the effort and reward associated with initiating a behavior, weighing the costs against potential benefits, and the premotor state of preparing an effortful action or action anticipation (Glimcher and Fehr 2013).

The way in which effort and reward influence decision-making has been extensively studied [for reviews, see Kable and Glimcher (2009); Rushworth et al. (2011); see also Shima and Tanji (1998); Hartmann et al. (2013)]. Physical effort costs have consistently been found to be reflected in sensorimotor integration areas of cingulate cortex, supplementary motor area (SMA), and the striatum (Croxson et al. 2009; Kurniawan et al. 2010; Prévost et al. 2010) whereas regions such as the nucleus accumbens (NAc) have been implicated in valuation of effort costs (Salamone et al. 2007; Botvinick et al. 2009; Salamone 2011). Some evidence supports the view that weighing up of costs versus benefits might in part be supported by the dorsal anterior cingulate cortex (dACC) (Walton et al. 2006; Rushworth and Behrens 2008; Hosokawa et al. 2013; Shenhav et al. 2013), whereas anticipation of effort production has been linked to SMA, cingulate motor areas (CMAs), and dorsal striatum (Walton et al. 2003; Cowen et al. 2012; Kurniawan et al. 2013). Consistent with the view that the computations performed in these regions might play a key role in motivation, lesions of either medial frontal cortex or the basal ganglia can lead to a profound state of pathological apathy in humans (Devinsky et al. 1995; Levy and Dubois 2006; Schmidt et al. 2008; Holroyd and Yeung 2012; Adam et al. 2013).

**Figure 1.**
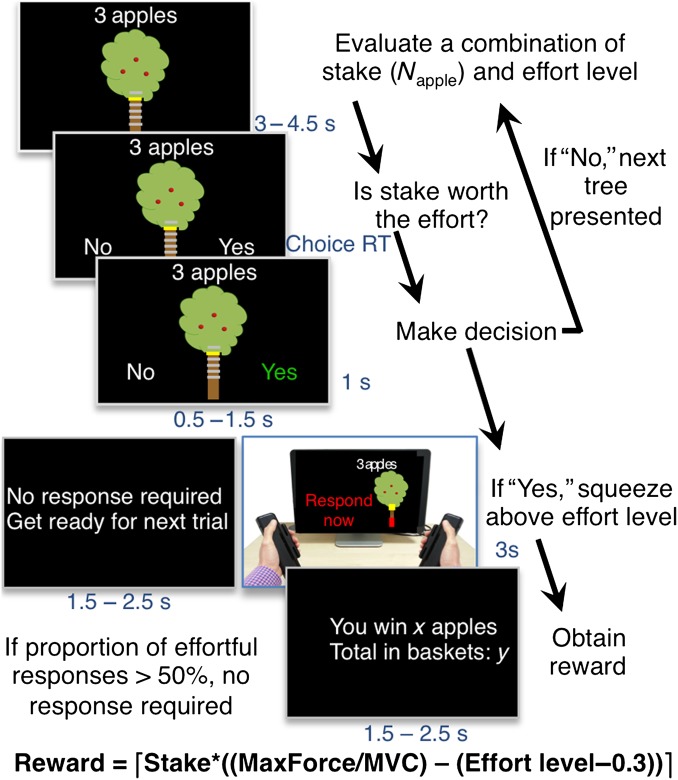
Effort- and reward-based decision-making task. Each trial starts with an apple tree showing the stake (number of apples) and effort level required to win a fraction of this stake (trunk height). There were 6 possible stakes (1, 3, 6, 9, 12, and 15 apples), and 5 possible effort levels (60%, 70%, 80%, 90%, and 100% of subject's MVC), indicated by the trunk height as well as yellow horizontal lines on tree trunk. After 3–4.5 s, participants decided whether or not they want to engage in an effortful response to gather apples (YES/NO option). Fruit gathering was performed by squeezing a force transducer with right or left hand, which translated on the screen as a red bar gradually filling the trunk. Subjects only won a percentage of the stake if they managed to reach or go beyond the top of the trunk. The expected reward was calculated on the basis of stake, effort level, and maximal force reached within 3 s. To control for the number of effortful responses produced, the selection of the YES option was also sometimes followed by a screen indicating that no response is required.

Here, our aim was to investigate which of the different processes discussed earlier is most critically involved in apathy observed in healthy individuals. We developed a paradigm to investigate effort- and reward-based decision-making (Bonnelle et al. 2014). In this simple computer game (Fig. 1), participants are presented on a trial-by-trial basis with an offer, which they can accept or reject. They are presented visually with a combination of a “stake” (or incentive) and an “effort” level. If they accept the offer, they are required to engage in an effortful physical response to obtain a “reward.” As in real-world situations, the actual reward obtained depends both on the stake on offer and on the physical force produced. If an individual rejects the offer, a new offer is presented.

This paradigm allows dissociation of several different processes occurring around the time the decision to engage in physical effort is made. The evaluation of “stake,” “effort,” and “expected reward” (estimation of potential outcome) as well as “cost-benefit weighing” may all be encoded in the brain before such a decision (Croxson et al. 2009; Rangel and Hare 2010; Hare et al. 2011; Rushworth et al. 2011; Kurniawan et al. 2013; Apps and Ramnani 2014). The decision to act then has to be converted into a motor plan for effortful action to take place (Rigoux and Guigon 2012). Willingness to exert effort on our task has previously been shown to be sensitive to individual differences specifically in behavioral apathy, indexed by the action initiation subscale of the Lille Apathy Rating Scale (LARS-e), modified for healthy people (Bonnelle et al. 2014).

We used functional magnetic resonance imaging (fMRI) to investigate whether apathy traits in young healthy volunteers relate to functional changes in any of the processes involved around the time of reward- and effort-based decision-making and action anticipation. In addition, we employed diffusion tensor imaging (DTI) to investigate how differences in white matter microstructure in pathways connecting regions functionally involved in evaluation, weighing or action anticipation might underlie interindividual difference in apathy traits. Finally, we performed an analysis of functional connectivity between these brain areas to determine whether there are also differences in the effectiveness of communication between them in apathetic people.

## Materials and Methods

### Participants

The study was approved by Oxford University Medical Sciences Inter Divisional Research Ethic Committee. All subjects volunteered for the study via a website and gave informed written consent before the study. Forty right-handed neurologically healthy participants with no current diagnosis of psychiatric disorder were recruited. In order to obtain a wide variation in the level of motivation, the second half of the participants we recruited were prescreened specifically for high behavioral apathy traits using a short questionnaire (15 questions) that comprised 6 questions taken from the Action Initiation subscale of a modified version of the Lille Apathy Rating Scale (see below), interleaved with 9 other questions unrelated to apathy so that potential participants were not aware of the prescreening selection criteria.

Two participants did not finish the experiment (one withdrew and another could not be scanned) and one was excluded due to a problem with the equipment during his participation. Thirty-seven subjects were therefore included in the reported analyses (17 males, mean age 26 ± 4.4, range 19–38, 25 students, 6 employed, and 6 unemployed). The study lasted 2 h, of which 1 h was in the MRI scanner. The monetary compensation participants received at the end of the experiment depended on their performance on the task (both outside and inside the scanner) and varied between £15 and 20.

### Questionnaires

Self-reports of apathy traits were obtained using a modified, extended version of the original Lille Apathy Rating Scale (LARS-e), available online in Bonnelle et al. (2014) (see Supplementary Material for more details). The LARS-e uses subscales that allow assessment of apathy traits along several domains reflecting the distinct component of apathy (behavioral, cognitive, and emotional). We used the “Action Initiation” (AI) subscale of the LARS-e, which measures every-day productivity and initiative and is an index of behavioral apathy (Sockeel et al. 2006). This subscale was previously found specifically to relate to the willingness to engage in an effort response in order to obtain a reward on our paradigm (Bonnelle et al. 2014). In addition, to control for a potential confound of depression and anhedonia, we also used the Depression, Anxiety, Stress Scales (DASS) (Lovibond and Lovibond 1995), a questionnaire developed in nonclinical populations to measure depression, and the Snaith–Hamilton Pleasure Scale (Snaith et al. 1995), which assesses anhedonia.

### Distribution of Apathy Traits in Participants

To determine a cut-off for “high” behavioral apathy traits in a normal young population sample, we combined data from all the experiments performed in our lab where the LARS-e has been administered (*N* = 139, mean age 27.4 ± 7.8). For ease of exposition, so that higher values indicate greater degrees of behavioral apathy, we took the AI scores and subtracted them from the score maximum of 5. The mean of the behavioral apathy distribution (5—AI score) was 1.46, with a 95% confidence interval comprised between 1.33 and 1.58. We therefore considered scores of >1.58 to reflect behavioral apathy. Of the 37 participants included in the present study, 16 met this criterion for apathy. In our group, individuals who scored above the cut-off were not significantly more depressed (DASS scores comparison, *P* = 0.5), although they were slightly more anhedonic (*t* = 2.14, *P* = 0.039). This is potentially interesting given the literature relating to motivation deficits in anhedonia (e.g., reviewed in Treadway and Zald 2011).

### Apparatus

Stimulus presentation was programed in MATLAB (The MathWorks, Inc., USA) using the Psychtoolbox (http://psychtoolbox.org). Force was recorded using a TSD121B-MRI hand dynamometer (BIOPAC Systems, Inc., USA) with a sample rate of 500 Hz. The recorded signal was digitalized and fed in real-time into the PC running the task program.

### Estimation of Maximal Voluntary Contraction

At the beginning of the experimental session, each participant's maximum voluntary contraction (MVC) was established so that effort levels were normalized across individuals. They were asked to squeeze the handles of the dynamometer as strongly as they could with their right and left hands. The maximum force was recorded for each hand. Subsequently, participants were instructed to squeeze each handle until the level indicating their force online went above a yellow bar, corresponding to 100% of the maximum force previously recorded for the same hand. If they managed to reach that level, the procedure was repeated using the new maximum, else the first value was kept. The procedure was repeated 3 times, and the maximal force reached was used as the MVC for that participant. To account for potential fatigue effects over time in the experiment proper, MVC was adjusted on each block so that it corresponded to 95% of the maximum force reached over the previous block. In addition, if a force higher than MVC was produced, the MVC would automatically be adjusted to this new value on subsequent trials.

### Task Description

Each trial started with the presentation of an offer: an apple tree that combined 1 of the 6 possible levels of stake (1, 3, 6, 9, 12 and 15 apples), and 1 of 5 possible effort levels (60%–100% of subject's MVC), indicated by the height of the tree trunk and highlighted in yellow on the top of the trunk (Fig. 1). Participants then had to decide whether or not they wanted to engage in an effortful response to win a percentage of the presented stake: that is, accept or reject the offer. They selected a “YES” or “NO” option by gently squeezing one of the handles (left or right) corresponding to the location of the option on the screen (the side was randomized across trials). If the tree was judged “not worth the effort”, they chose the “NO” option and the trial ended. On the other hand, if the “YES” option was selected, the tree reappeared on the left or right of the screen, indicating which hand should be used for the execution of the effortful response. The side of the effortful response was also random and independent of the accept/reject sides. Subjects were therefore unaware of which hand would have to be used for the effortful response at the time of the decision.

Direct on-line visual feedback of the force exerted—with respect to the level required to obtain the reward—was provided by a red bar that filled the trunk as participants squeezed the handgrip device. Participants had 3 s to squeeze the dynamometer handle to move the red bar above the top of the trunk (Fig. 1). If they failed to reach the top of the trunk, no apples were gathered. The number of apples gathered (and therefore the reward accumulated) was otherwise determined as follows:

(1) $$\text{Reward}=\text{Stake}\times \left(\left(\frac{\text{MaxForce}}{\text{MVC}}\right)-(\text{Effort}\;\text{level}-0.3)\right).$$

MaxForce corresponds to the maximum force reached over the 3-s response period, whereas MVC is the MVC established prior to the experiment. Note that with this reward function, participants could win “a percentage of the stake” (number of apples) once they reached the required effort level (0.3 is an arbitrary term used so that a positive reward is obtained when subjects just manage to reach the required effort level). However, if after achieving this level, they exerted more force, they were able to increase the “reward” obtained (yield of apples) further. Thus, the reward obtained for a given physical exertion depended nonlinearly on the trial's stake and effort level. Participants were instructed to gather as many apples as they wanted over the course of the experiment, knowing that the money they would receive at the end would depend on the total number of apples gathered during the experiment (minimum: £15, maximum: £20).

Participants were instructed to perform the task as intuitively as possible, by reproducing a behavior they would have in real-life fruit gathering, where the higher a tree trunk is the less easily accessible the fruits are and therefore the harder it is to collect them (high effort level associated with lower gathering yield). It was also explained that the reward they obtain depends on the effort they engage in, just as in real-life gathering. This minimized any interindividual difference due to learning (see Supplementary Material). Participants were trained on these different levels at the beginning of the experiment, so that they had experience of the physical force required for each effort level when they started the task. In addition, although they were not explicitly told so, the first 16 trials were considered as training and not included in the analysis. Participants performed 4 blocks of the task in the scanner. Each block consisted of 40 trials pseudo-randomly sampling the whole effort/stake space. Each effort/stake combination was presented 5–6 times. In order to efficiently model choice behavior, they also performed 2 blocks of the task outside the scanner (see Supplementary Material). To reduce fatigue effects, outside the scanner blocks were interleaved with questionnaires, whereas inside the scanner structural scans or field maps were used to break up test blocks. In addition, we ensured that different participants actually performed the same number of effortful responses (see Supplementary Material—Task description).

### Behavioral Analysis

The decision to engage in effort may depend on a number of situational variables. First, if higher efforts are required to obtain a reward then, for a given amount of effort exertion, the “expected reward” is lower. This is captured by our reward function (eq. 1). Second, exerting effort might itself constitute a cost to the participant. Third, a participant might not be successful in producing higher effort levels, so there might be important effects of probability of success on decision-making, with greater uncertainty and less likelihood of being successful when the effort required is higher. Fourth, the stake on offer might potentially motivate an action, independently of its effect on reward expectation. An important aspect of our choice of reward function is that it allows dissociation of the respective contributions of these 4 factors. Participants' choices were thus modeled using a logistic function, which allows dissociation of the respective impact of “expected reward” (gathering yield), “effort level” (tree height), “probability of success,” and “stake” (number of apples) on choice.

We first calculated “expected reward”: the reward a participant would obtain for exerting a particular force, given the combination of stake and effort level on offer. This was estimated from equation (1), for a maximum force corresponding to the MVC (i.e., Expected reward = [Stake × (1.3 − Effort level)]^+^). Since the expected reward includes an interaction between stake and effort, this provides an opportunity to examine effects of the stake cue and effort-level cue independently of expected reward. The 3 terms (stake, effort, and stake × effort) can be written in terms of expected reward, with further contributions from stake and effort levels. This gives “Stake′” = {stake + αReward} and “Effort′” = {effort + βExpected Reward}, orthogonalizing stake and effort with respect to expected reward by linear regression, with α and β corresponding to the slopes of the regressions. This orthogonalization quantifies the effects of the stake and effort cues independently of how much reward could be obtained for a given stake and effort level. Thus, considering these effects (“Stake′” and “Effort′”) separately enabled us to dissociate the respective contribution of stake cues and effort cues from expected reward.

We also accounted for the “probability of success” (i.e., the probability of successfully reaching the top of the trunk given the effort requirement) based on all previous trials (including those outside the scanner) since a lower chance of obtaining reward at high effort levels might independently contribute to the subjective value of an option. This was orthogonalized with respect to effort using linear regression (*P*_success_′). These 4 variables were demeaned and normalized before being entered in the logistic regression model:

(2) $$\begin{array}{cc} P(\text{yes}) & =\frac{1}{(1+\text{exp}-(\beta_{0}+\beta_{S}\times {\text{Stake}}^{\prime }+\beta_{E}\times {\text{Effort}}^{\prime }+\beta_{R}}\\ & \;\;\;\times \text{Reward}+\beta_{\mathrm{PS}}\times {P_{\mathrm{success}}}^{\prime })) \end{array},$$

where *β*_0_ is the response bias parameter and *β*_S_, *β*_E_, *β*_R_ and *β*_PS_ are subject-specific choice parameter estimates respectively characterizing the impact of incentive salience (Stake′), effort requirement (Effort′), expected reward and probability of success on choice.

### MRI Acquisition

We used a 3Tesla Siemens MRI scanner (maximum gradient strength, 40 mT m^−1^) with a four-channel Nova birdcage coil to collect T2*-weighted echoplanar images (EPIs) (45 × 3 mm slices; repetition time [TR], 3.0 s; echo time [TE], 30 ms; matrix, 64 × 64 voxels; field of view, 192 × 192 mm). We used a slice angle of 15° from the horizontal plane for optimizing scans of orbital and ventral frontal brain regions. A T1-weighted FLASH image was acquired for each subject (TR, 2040 ms; TE, 4.7 ms; flip angle, 90°; voxel size, 3 × 1 × 1 mm).

Diffusion-weighted volumes with gradients applied in 64 noncollinear directions were collected. The following parameters were used: 64 contiguous slices, slice thickness = 2 mm, FOV = 192 mm, matrix = 128 × 128 mm, TR = 8900 ms, TE = 91.2 ms, voxel size = 2 mm isotropic, acquisition 6/8 partial Fourier, 60 diffusion directions with *b*-value = 1500 s/mm^2^, and 4 images with no diffusion weighting (*b* = 0 s/mm^2^), bandwidth = 1680 Hz/pixel. Head motion was minimized by the use of tightly padded clamps attached to the head coil.

### Functional MRI Analysis

#### Preprocessing

Analysis was performed using tools from the software library of the Oxford Centre for Functional Magnetic Resonance Imaging of the Brain (FMRIB) (http://www.fmrib.ox.ac.uk/fsl). We discarded the first 2 fMRI volumes to allow for T1 equilibrium effects. We performed probabilistic independent components analysis on the rest of the images to identify and remove large motion artifacts (Beckmann and Smith 2004). We selected manually and conservatively the components that clearly appeared as noise only (movements, cardiac or respiratory artifacts, http://fsl.fmrib.ox.ac.uk/fslcourse/lectures/practicals/melodic/). We corrected the ICA-adjusted data for motion (Jenkinson et al. 2002). The data in each volume were spatially smoothed with a 6-mm full-width half-maximum Gaussian kernel. We applied a high-pass temporal filter of 100 s to the data to remove low-frequency noise that may arise from scanner drift. EPI images were unwarped with field maps to improve the registration. Images were skull-stripped and then coregistered using FMRIB's linear registration tool, each subject's EPI images being registered with their high-resolution structural image and transformed into standard space (Montreal Neurological Institute [MNI]) using affine transformations.

#### First-Level Analysis

FMRI data were analyzed using voxel-wise time series analysis within the framework of the General Linear Model (GLM). To this end, a design matrix was generated with a synthetic hemodynamic response function (gamma convolution, phase = 0 s, Stdev = 3 s, mean lag = 6 s) and its first temporal derivative. Several types of events were distinguished. To tease apart the different processes taking place during the choice period, 6 distinct explanatory variables (EVs) were modeled. The same 4 decision variables as the ones used for the choice modeling were used to model expected reward, effort requirement, stake, and probability of success. The probability of each participant being willing to engage (*P*[yes]) was estimated for each stake and effort combination using the choice model (see equation [2]). Similarly, each participant's cost-benefit weighing load was computed as |*P*yes − 0.5|, which was maximal when *P*(yes) was close to 50% and minimal when close to 0% or 100%.

These EVs were modeled as epochs of variable duration that took into account choice reaction-times (i.e., from stimulus onset to the time the choice response is being made) (Grinband et al. 2008).

Cost-benefit weighing load and *P*(yes) were orthogonalized in FSL with respect to the decision variables. The probability of being willing to engage, *P*(yes), once orthogonalized with respect to all the variables weighing on the decision process (stake, effort, expected reward, and probability of success) can be considered as a postdecisional variable reflecting the level of action anticipation.

Two additional EVs were added to the GLM to model the effortful response period and the reward period. The effort period was modeled as an epoch lasting 3 s (response window) and was parametrically adjusted on a trial-to-trial basis to reflect the effort actually exerted (Force/MVC). Similarly, the reward period was modeled as an epoch lasting the time of the reward presentation and was parametrically adjusted to reflect the reward obtained. Our GLM design was estimated as efficient in FSL FEAT, with effects-size required lower than 2.2% (Supplementary Fig. 1).

We used cluster-based thresholding (clusters determined by *Z* = 2.3 and a significance threshold of *P* = 0.05 corrected for multiple comparisons [Worsley et al. 1992], as in Croxson et al. [2009]).

#### Higher-Level Analysis

The 4 blocks were first combined using fixed-effects analysis. Higher-level analysis was then performed using FMRIB's local analysis of mixed effects to investigate the group average. We also used a multiregression model to investigate the relation between apathy traits, choice parameters, and BOLD signal change. Depression and anhedonia scores were added as regressors to dissociate these from apathy. Final statistical images were thresholded using Gaussian Random Field-based cluster inference with a height threshold of *Z* > 2.3 and a cluster significance threshold of *P* < 0.05.

#### Region of Interest Analysis

To further explore the change in activation in different regions of the medial wall, a region of interest (ROI) analysis was performed, using connectivity-based defined masks generated as in Beckmann et al. (2009) (courtesy of M. Rushworth) for the pre-SMA, the SMA, M1, the dorsal ACC (Brodmann area 32d), and 3 subdivisions of the CMA (anterior and posterior rostral cingulate zones and caudal cingulate zone) (Fig. 4*e*). Mean percentage BOLD signal change was extracted from these masks. Nucleus accumbens ROI was generated using the Harvard–Oxford subcortical structural atlas provided by the Harvard Center for Morphometric Analysis.

#### Functional Connectivity Analysis

Psychophysiological interaction (PPI) analysis is a method for investigating task-specific changes in the functional connectivity in different brain areas (O'Reilly et al. 2012). We used this method to investigate individual differences in functional connectivity between the SMA and the rest of the brain during response anticipation (Supplementary Fig. 3), and how this may relate to apathy traits (Fig. 7*a*). The interaction term used as regressor in the first-level analysis GLM is the scalar product of the task time-course for the periods of interest—here, the choice period for accepted trials—and the physiological time-course (time-course of activity in the SMA mask). The resulting map shows regions where the BOLD signal on accept trials correlated more positively with the signal in the SMA, relative to baseline. Apathy scores were added as regressor for the group-level analysis to investigate regions that were more or less connected to the SMA during motor response preparation as apathy scores increased.

### Diffusion Tensor Imaging Analysis

#### Preprocessing

Diffusion-weighted images were registered to the *b* = 0 image by affine transformations to minimize distortion due to motion and eddy currents and then brain-extracted. Voxel-wise fractional anisotropy (FA) maps were generated using FDT in FSL (Behrens, Johansen-Berg, et al. 2003).

#### Tract of Interest Analysis

The following tracts, from the JHU White-Matter Tractography atlas available in FSL, were used: the cingulum bundle, the anterior thalamic radiation, the superior longitudinal fasciculus, and the cortico-spinal tract (right hemisphere). In addition, we generated a mask for the white matter pathway connecting the dorsal striatum to the SMA using tractography (see below). Indeed, these 2 brain regions have frequently been found to be involved in effort evaluation and anticipation (Romo and Schultz 1992; Kurniawan et al. 2013) and have been found to be structurally (Lehéricy et al. 2004) and functionally connected (Martino et al. 2008).

In an approach similar to Bonnelle et al. (2012), the tracts of interests were projected into each individual's diffusion tensor imaging space. The obtained maps were binarized and applied to the FA maps to obtain one mean FA value per tract and per subject. Mean FA values were thus calculated from the area of overlap between the whole white matter skeleton and the mask of the particular tract in individual space. We then used linear regression to derive FA values corrected for any effects of age in the analyses reported. In addition, to control for nontract-specific effects, mean FA within each tracts was also regressed out for whole white matter skeleton mean FA.

#### Tractography

To generate a mask for the tract connecting the dorsal striatum to the SMA, individual tractography was performed in subgroup of 10 subjects randomly selected among our 37 subjects using probabilistic tractography in FSL (Behrens, Woolrich, et al. 2003). 5-mm and 10-mm-radius spherical ROIs were created for the Putamen and the SMA, respectively. The coordinates for these masks corresponded to peak of maximal signal intensity in the fMRI group analysis for *P*(yes)-related signal (posterior putamen [28, −12, 6] and SMA [4, −4, 52]). Two tractographies were performed using each of these masks as seed or termination point (i.e., A to B and B to A). Individual tractography outputs were brought to the standard space using nonlinear transformations. The projected tracts were then averaged. The 2 tracts generated (A to B and B to A) were then overlaid, thresholded, and binarized. The resulting tract was used as mask for the tract of interest analysis presented earlier.

## Results

### Behavioral Choice Modeling

**Figure 2.**
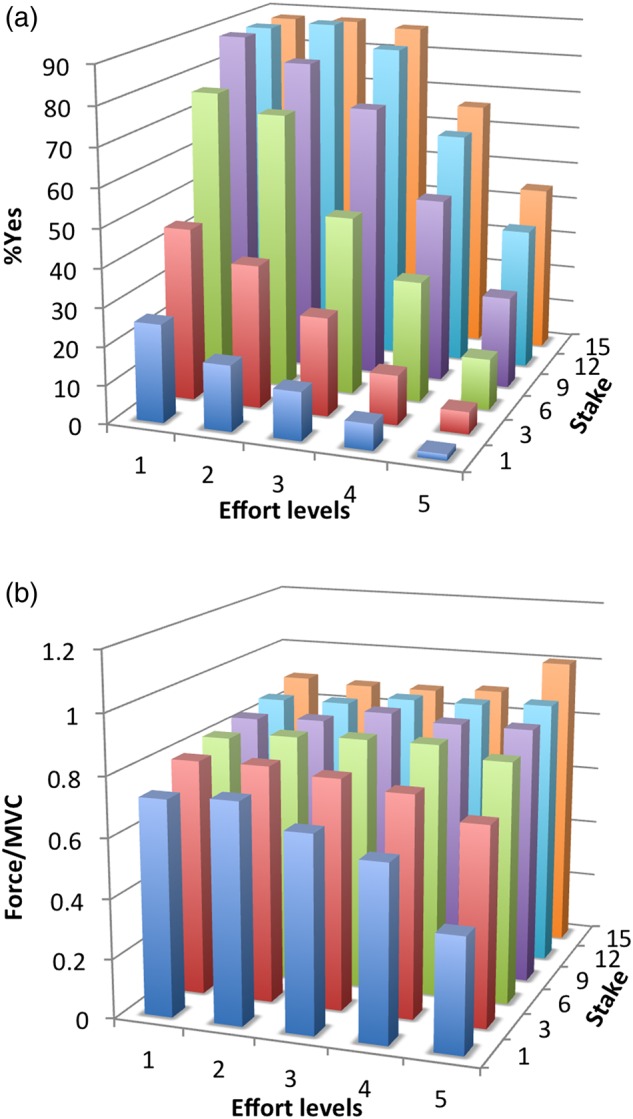
Behavior on task. (*a*) Percentage of accepted trials (%Yes) and (*b*) force exerted relative to MVC averaged across participants plotted against effort levels and stakes. Effort levels (1–5) correspond to percentage of MVC (from 60 to 100% MVC).

Stake (number of apples) and effort cues both had a significant impact on participant's choices (Fig. 2*a*, repeated-measures ANOVA stake × effort: effect of stake cue *F* = 102.9, *P* < 0.0005; effect of effort cue *F* = 80.6, *P* < 0.005; interaction stake × effort *F* = 3.3, *P* = 0.008). Logistic regression quantified the contributions of stake, effort requirement and expected reward on choice. Since expected reward included a stake × effort interaction, the effect of stake and effort cues could be orthogonalized with respect to expected reward (see Materials and Methods). This orthogonalization quantifies the effects of the stake and effort cues independently of how much reward could be obtained for a given stake and effort level. In addition, we controlled for each participant's probability of success at a given effort level. To do this, the probability of success based on previous trials was included as a regressor, so that it would not confound measurement of “effort sensitivity” (impact of effort on choice).

**Figure 3.**
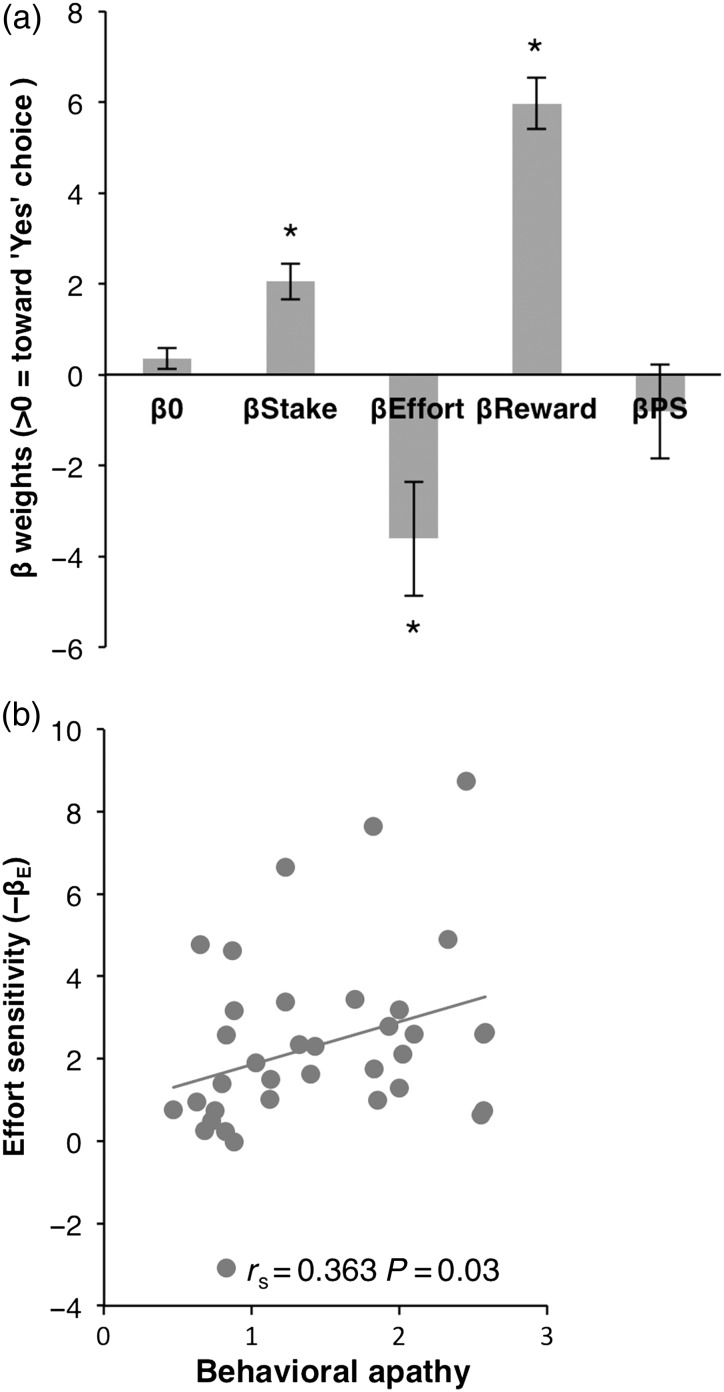
Choice probability modeling and relation with apathy traits. (*a*) Mean response bias (*β*_0_) and beta weights for stake, effort, expected reward, and probability of success across participants. Positive values indicate a weight toward “Yes”. *One-sample *t*-test, *P* < 0.05. (*b*) Correlation between behavioral apathy scores and effort sensitivity (*β*_Effort_).

Stake′, effort requirement, and expected reward all had a significant impact on choice (Fig. 3*a*; one-sample *t*-tests, *β*, est′: *t* = 4.95, *P* < 0.0005; *β* Effort′: *t* = −6.20, *P* < 0.0005; *β* ExpReward: *t* = 10.68, *P* < 0.0005). There was no significant response bias (*β*_0_: *t* = 1.61, *P* = 0.11) or any significant effect of the probability of success based on previous trials (*β P*_success_ *t* = 0.86, *P* = 0.39) (see Supplementary Table 1 for individuals' model parameter estimates).

As might have been expected “effort sensitivity” (i.e., *β* effort′ was significantly related to behavioral apathy trait (*r* = 0.363, *P* = 0.03) (Fig. 3*b*). No significant correlation was observed between behavioral apathy and stake sensitivity (*r* = −0.293, *P* = 0.08) or expected reward sensitivity (*r* = −0.193, *P* = 0.26) (Supplementary Table 2). Thus, more apathetic individuals were more sensitive to effort than more motivated participants (Fig. 3*b*). There was no significant correlation between behavioral apathy scores and percentage of yes/no choices, choice response times, or overall force exerted during effortful responses (Supplementary Table 2).

### Distinct Brain Processes Involved during Decision-Making

**Figure 4.**
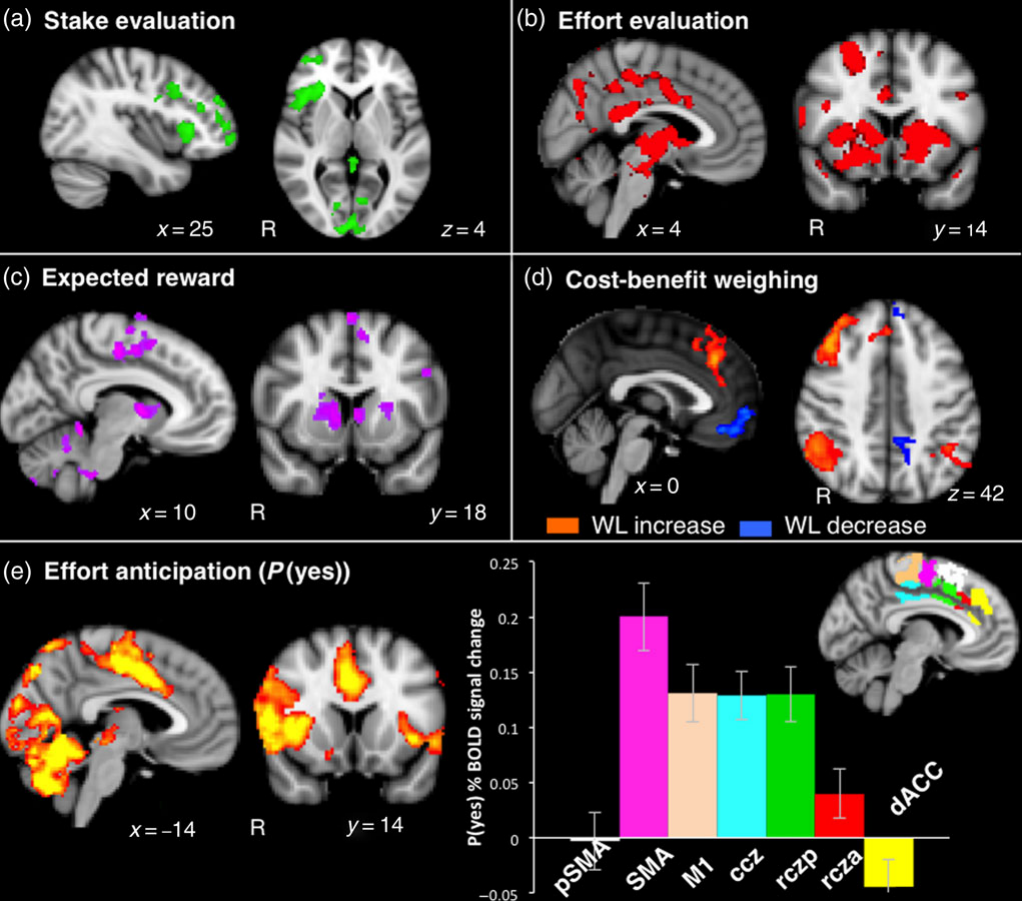
Brain regions associated with behavioral processes during the decision period. Regions showing significant increase in activation with (*a*) stake increase, (*b*) effort decrease, (*c*) expected reward increase, (*d*) cost-benefit weighing load (WL) with increase in orange and decrease in blue, and (*e*) increased probability of willing to engage effort, that is, probability of responding YES. Right panel shows BOLD signal increase with probability of accepting an offer for different medial frontal regions (±standard error), parceled out based on connectivity for pSMA), SMA, primary motor cortex (M1), caudal cingulate zone (ccz), posterior, and anterior rostral cingulate zones (rczp; rcza).

Each of the processes of interest could be dissociated in the fMRI analysis (see Materials and Methods). “Expected reward,” that is, how much reward could be obtained for a given physical force, correlated with increased activation in the caudate as well as CMA and SMA (Fig. 4*c*, Supplementary Table 3). “Effort evaluation” was associated with change in activation in the basal ganglia (nucleus accumbens, caudate, putamen) as well as the CMA (Fig. 4*b*, Supplementary Table 3), so that an increase in effort requirement produced a decrease in activation. “Stake evaluation” activated right ventrolateral frontal regions often implicated in directing attention (Corbetta and Shulman 2002) (Fig. 4*a*, Supplementary Table 3). Finally, “cost-benefit weighing”—which becomes more difficult as expected costs come close to possible benefits—was positively correlated with activation in a network of brain regions previously been associated with cognitive control (Botvinick et al. 2001; Kerns et al. 2004; Nachev et al. 2005, 2008) on the medial frontal wall, including dACC and pre-SMA, and negatively correlated with activation in ventromedial prefrontal cortex (Fig. 4*d*, Supplementary Table 4).

### Anticipation of Effort and Response Preparation

The probability of being willing to engage in an effortful response (*P*[yes]), when considered orthogonally to option value characteristics (effort, stake, and expected reward), can be viewed as reflecting response anticipation or response preparation following the decision. The activation pattern associated with this regressor demonstrated extensive activation in premotor-sensorimotor regions such as CMA, SMA, and primary motor cortex (M1), consistent with this view (Fig. 4*e*, Supplementary Table 4).

It is unlikely that this signal reflects the action needed to select the YES/NO options as all trials are associated with a choice motor response, regardless of the willingness to subsequently engage in an effortful action. In addition, we accounted for trial-to-trial differences in choice reaction-times (and potentially associated shift in the hemodynamic response) by modeling EVs of variable epochs based on the choice duration (see Material and Methods).

This signal was not associated with activation during the effortful response either, as the same pattern of BOLD signal change was observed when including only the trials that were not followed by an effortful response period (“NO” trials and “No response required” trials; Supplementary Fig. 2*a*) in the fMRI analysis.

An ROI analysis was performed to further characterize recruitment of the medial frontal wall regions for this last process. SMA, CMAs (posterior rostral cingulate zone, rczp, and caudal cingulate zone, ccz), and primary motor cortex (M1) all showed a significant increase in BOLD signal with increased probability of accepting an offer (Fig. 4*e* right), consistent with greater activity in anticipation of an effortful motor response (Shima and Tanji 1998; Kurniawan et al. 2013).

### Behavioral Apathy Scores and Effort Anticipation

**Figure 5.**
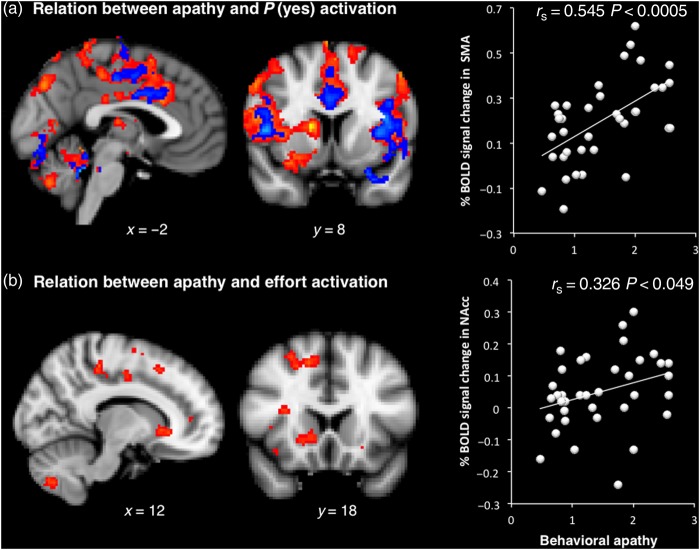
Relationship between brain function and individual differences in apathy traits. (*a*) Whole-brain correlation between apathy scores and BOLD signal increase with increased probability of accepting an offer controlling (blue–light blue) or not (yellow–red) for variance explained by behavioral model parameters (effort, stake, and reward sensitivity). Top right panel shows correlation between behavioral apathy scores and activation increase with *P*(yes) in the SMA. (*b*) Whole-brain correlation between apathy scores and effort-related BOLD signal change (signal increase with decreased effort). Bottom right panel shows relation between behavioral apathy scores and activation increase (as effort level decreased) in the nucleus accumbens (NAc).

Importantly, “behavioral apathy” scores were strongly correlated with signal change in several of the regions involved in response preparation/anticipation, as *P*(yes) increased (SMA: *r*_s_ = 0.545, *P* < 0.0005; rczp: *r*_s_ = 0.469, *P* = 0.003; ccz: *r*_s_ = 0.472, *P* = 0.003; and M1: *r*_s_ = 0.458, *P* = 0.004; Fig. 5*a*—yellow–red activation map, Supplementary Table 5). In line with our hypotheses and previous work (Bonnelle et al. 2014), there was no correlation with emotional or cognitive apathy in those regions. Thus, it appears that individuals who were more apathetic had to recruit more neural resources in anticipation of execution of an effortful action.

This effect could not be explained by a difference in choice response times, as these were not correlated with apathy scores (see Supplementary Table 2). Crucially, it was not related to movement during effort production, because the same result was obtained when only including trials that were not followed by a response (Supplementary Fig. 2*b*). The result also remained significant after controlling for individual differences in stake, effort, and reward sensitivity choice parameters (Fig. 5*a*—blue activation map), as well as variance of probability of responding YES (Supplementary Fig. 2*c*). We also controlled for interindividual differences in the quality of the behavioral model fit. The correlations between “behavioral apathy” scores and signal change as *P*(yes) increased were still highly significant after controlling for individuals akaike information criterion (partial correlations, SMA: *r* = 0.562, *P* < 0.0005; ccz: *r* = 0.555, *P* < 0.0005; and M1: *r* = 0.489, *P* = 0.002).

### Behavioral Apathy Scores and Effort Evaluation

Individuals with higher behavioral apathy also showed less activation in the nucleus accumbens, SMA, and mid-cingulate cortex (including dACC as well as rczp, rcza, and ccz) as effort level increased (Fig. 5*b*). This pattern of recruitment of regions associated with effort discounting (devaluation)(Walton et al. 2006; Botvinick et al. 2009; Croxson et al. 2009) would be consistent with elevated sensitivity to effort observed behaviorally in more apathetic individuals (Fig. 3*b*). Indeed, when both apathy scores and subject-specific effort sensitivity parameters (*β*_Effort_) were added in a whole-brain multiregression analysis, the latter explained most of the interindividual effort-related BOLD signal variance, whereas the relation with apathy scores no longer produced significant activation map.

### Apathy Traits and Structural Connectivity

We next investigated whether integrity of white matter pathways connecting the brain regions involved during the task could predict individual differences in apathy. Fractional anisotropy measures within the 5 tracts of interest (see Materials and Methods) were entered in a binary logistic regression model aimed at classifying participants into those with high and low behavioral apathy (median split). Only the cingulum bundle was selected in a model that could correctly classify the subjects with 74.3% of accuracy (69% for high apathy and 79% for low apathy) (chi-square = 11.24, df = 1, *P* = 0.001).

**Figure 6.**
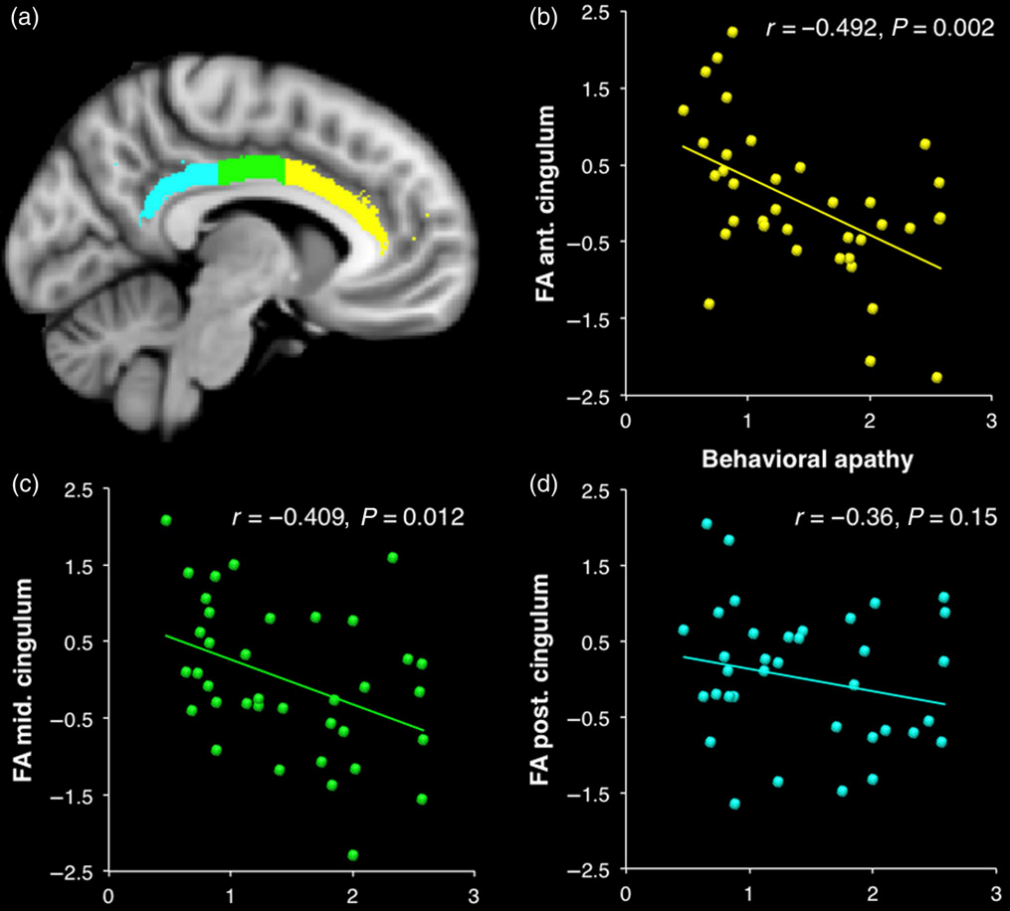
Relationship between cingulum white matter structure and apathy traits. (*a*) Cingulum bundle mask was parceled into 3 portions: anterior (yellow), middle (green), and posterior (blue). Correlations between behavioral apathy scores and normalized mean FA corrected for age and whole-brain white matter mean FA are plotted for the anterior (*b*), middle (*c*), and posterior (*d*) portions of the cingulum bundle (bilaterally).

Spearman correlations confirmed that only the mean FA of the cingulum bundle showed a strong relationship with apathy traits (*r*_s_ = −0.500, *P* = 0.002). This tract contains association fibers with patterns of connectivity along its rostrocaudal extent, mirroring functional segregation along the cingulate gyrus (Vogt et al. 1992; Paus 2001; Beckmann et al. 2009). When anterior, middle, and posterior segments of the cingulum (Jones et al. 2006) (Fig. 6) were separately assessed, there was a gradient, with the anterior portion most strongly related to behavioral apathy scores (*r*_s_ = −0.492, *P* = 0.002), the middle portion less (*r*_s_ = −0.409, *P* = 0.012), and the posterior portion not at all.

We next investigated the relation between white matter structure in the cingulum and brain activation during action preparation. We first looked at the correlations between SMA activation and FA measures within the 3 portions of the cingulum bundle investigated (anterior, middle, and posterior). The middle portion appeared to be the most significantly correlated with SMA activation (*r* = −0.45, *P* = 0.015, Bonferroni corrected). Importantly, the correlation was still significant after controlling for behavioral apathy scores, which are strongly correlated with both measures (partial correlation coefficient: −0.357, *P* = 0.033).

### Apathy Traits and Brain Functional Connectivity

If the structural integrity of connections between medial frontal regions is abnormal in apathetic individuals, we would also expect there to be decreased functional connectivity between these areas. A PPI analysis was therefore performed, seeding from SMA (the area most strongly correlated with increased activity associated with willingness to respond in apathy, Fig. 4*e* right) specifically during the “choice period” for trials where participants were willing to engage effort. On average, SMA activity during choices where an effortful response is anticipated (i.e., “yes” trials) correlated with activity in M1, mid-cingulate and ACC, bilateral inferior frontal junctions, frontal eye fields, and intraparietal sulci (Supplementary Fig. 3 and Supplementary Table 6). Correlated activity was also observed in the striatum (caudate and putamen) and thalamus. Anticorrelated activity was found in the precuneus and bilateral inferior parietal cortices as well as in the occipital cortex.

**Figure 7.**
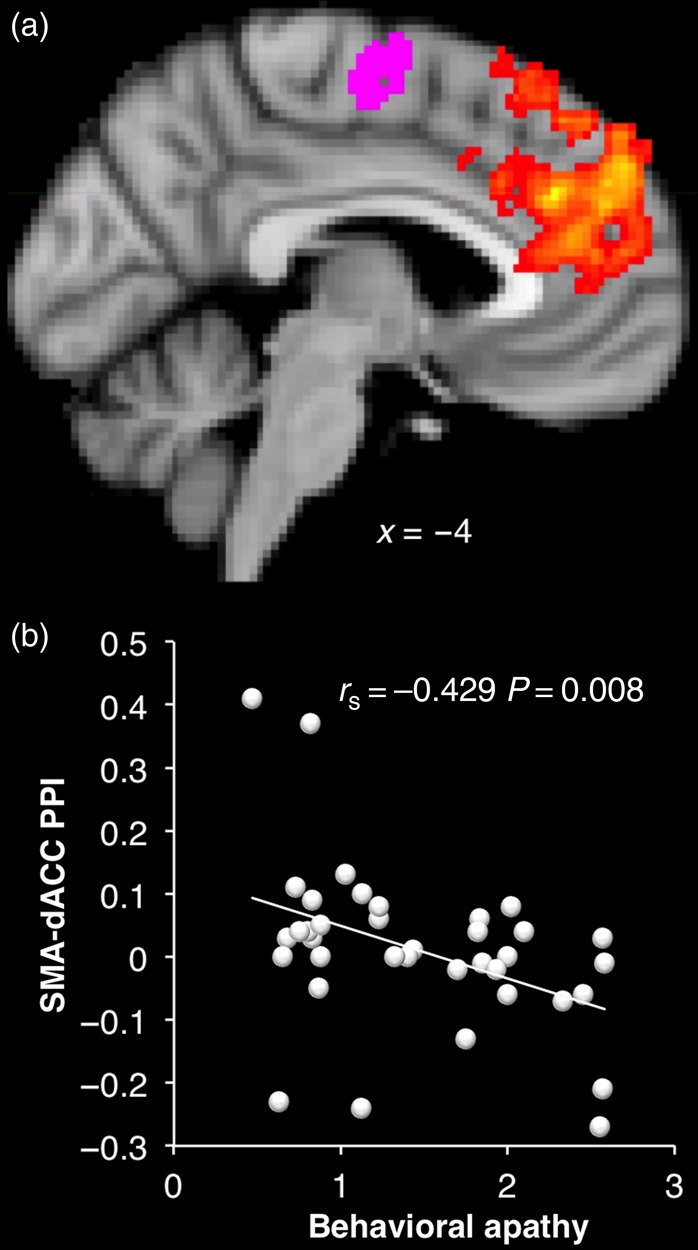
Relation between SMA functional connectivity and apathy traits. (*a*) In yellow–orange, regions where activity during the decision period on YES trials is more strongly correlated with activity in the SMA (purple) in more motivated individuals. (*b*) Correlation between behavioral apathy scores and the strength of the correlation (or functional connectivity) between the SMA and the dorsal ACC.

A whole-brain regression analysis revealed a significant correlation between functional connectivity with the SMA during choice periods of accepted trials and behavioral apathy scores in anterior and posterior cingulate regions, including the dACC (Fig. 7), overlapping with the region identified for cost-benefit weighing previously (Fig. 4*d*). Individuals with more behavioral apathy had less functional connectivity between these 2 medial regions.

## Discussion

Is there a biological basis to apathy? The findings presented here suggest there might be. We investigated whether behavioral apathy traits in the healthy population are associated with differences in the recruitment and structure of the neural systems involved in effort- and reward-based decision-making. We used a task that allowed dissociation of different processes involved around the time of decision: stake, effort and reward evaluation, cost-benefit weighing, and anticipation or preparation of effort production. Performance on this task allowed us to distinguish different brain systems involved in each of these processes in our healthy population (Fig. 4).

At a behavioral level, increased sensitivity to physical effort was observed in more apathetic individuals (Fig. 3). At the neural level, this was associated with greater recruitment of regions previously associated with effort discounting such as the nucleus accumbens (Botvinick et al. 2009; Kurniawan et al. 2010; Salamone 2011) (Fig. 5*b*). Paradoxically, increased recruitment of neural resources at the response preparation level was also observed in more apathetic people, particularly in mid-cingulate and premotor regions of the medial frontal wall, known to be involved in anticipation of effort production and action preparation (Walton et al. 2003; Prévost et al. 2010; Cowen et al. 2012; Kurniawan et al. 2013) (Fig. 5*a*). Finally, there was a strong relationship between apathy and both structural and functional connectivity between anterior and mid-posterior regions of the medial wall (Figs 6 and 7).

The strongest relationship between apathy traits and brain function was observed with the regressor indexing the probability of being willing to engage in an effortful response (Fig. 5*a* right panel). This regressor was made independent of putative upstream processes such as effort or reward evaluation and weighing. We therefore characterize this as a postdecisional process not related to the value of the proposition, but to the planning or anticipation of the response: the higher the probability of accepting an offer was, the more likely motor preparation in anticipation of the forthcoming effortful response. In keeping with this, at the neural level, this regressor covaried with signal in a network of cortical and subcortical areas associated with planning or even the urge to make a movement, for example, SMA and CMA (Grafton et al. 1992; Winstein et al. 1997; Prut and Fetz 1999; Jackson et al. 2011; Draper et al. 2014).

The increased activation for response anticipation/preparation in more apathetic individuals occurred in the absence of any difference in motor execution itself (no correlation between apathy scores and force production). Furthermore, it was evident after controlling for interindividual differences in stake, effort, and reward sensitivity. Although intriguing such an effect might reflect either a neural or a behavioral change (Price and Friston 1999). In other words, it might be either cause or effect of apathy. Increased neural inefficiency with higher apathy traits could imply elevated “physiological costs” of action initiation, with a need to recruit more brain resource to perform at the same level as more motivated individuals, thereby increasing effort sensitivity. Alternatively, this increase in BOLD signal might be due to higher “subjective experience” of effort cost in individuals who are more apathetic.

Increased SMA activation has indeed been observed in the preparation of more difficult tasks (Kurniawan et al. 2013), and transcranial magnetic stimulation of SMA leads to reduced perception of physical effort (Zénon et al. 2015). Medial frontal regions, including the SMA, have also been implicated in the urge for action (Jackson et al. 2011; Draper et al. 2014). However, the relation between apathy scores and BOLD signal change in medial frontal regions remained after controlling for subject-specific behavioral parameters such as sensitivity to effort, reaction time, or force produced, suggesting the possibility of a primary underlying neural, rather than psychological, cause. To investigate this possibility further, we also asked whether behavioral apathy might involve differences in underlying connections between decision and action preparation areas.

A recent, detailed postmortem dissection study of human brains demonstrated that fibers in the cingulate sulcus connect cingulate regions to the SMA (Vergani et al. 2014). In our analysis, cingulum bundle integrity, especially of the anterior portion, appeared as a strong structural predictor of individual's behavioral apathy traits (Fig. 6). Some studies in different clinical populations have reported evidence for a relation between structural integrity of this tract and “pathological” apathy in brain disorders (Cacciari et al. 2010; Ota et al. 2012; Tighe et al. 2012; Hahn et al. 2013). However, to our knowledge, the relation between white matter integrity and individual differences in apathy traits in the normal population has never previously been demonstrated.

The cingulate cortex has been proposed to play a crucial role in motivation by “energizing” action or task engagement (Stuss and Alexander 2007) and has been associated with allocation and adjustment of control based on task demands (Paus 2001). Lesions or gray matter volume loss here has been linked with pathological apathy (Apostolova et al. 2007; Kostić and Filippi 2011; Stuss 2011; Stanton et al. 2013). The recently proposed Expected Value of Control theory proposes that the output of dACC, which needs to be conveyed to premotor and motor regions (such as those identified in Fig. 4*e* right) to prepare and initiate an action, specifies the amount of control that is judged to be worth the expected reward (Shenhav et al. 2013). In keeping with this, strong reciprocal functional connections between the SMA and the mid-cingulate cortex have been observed during movement preparation (Nguyen et al. 2014). Impaired information flow between these systems may therefore affect the efficiency of the control exerted by cingulate regions, resulting in difficulty in action initiation. Our last analysis used PPI to confirm this prediction, with decreased functional connectivity observed between the SMA and the dACC in more apathetic individuals (Fig. 7).

The findings reported here demonstrate functional and structural markers underlying individual differences in behavioral apathy in healthy people. We speculate that decreased structural integrity of the anterior cingulum might be associated with suboptimal communication between key nodes involved in action energization and preparation, leading to increased physiological cost—and increased effort sensitivity—to initiate action. Thus, differences in motivation to act in healthy people might be due to differences in the brain's premotor control network.

## Supplementary Material

Supplementary material can be found at: http://www.cercor.oxfordjournals.org/.

## Funding

This research was supported by a Wellcome Trust Principal Research Fellowship to M.H. Funding to pay the Open Access publication charges for this article was provided by The Wellcome Trust.

## Notes

We thank Matthew Rushworth for kind donation of medial frontal region masks. *Conflict of Interest*: None declared.

## Supplementary Material

Supplementary Data
