# Supplementary material for: Individual Differences in Premotor Brain Systems Underlie Behavioral Apathy
Source: Cereb Cortex. 2015 Nov 12;26(2):807–19. doi: 10.1093/cercor/bhv247 (PMC4712805; doi:10.1093/cercor/bhv247)
Supplement: Supplementary Data [file supp_26_2_807__index.html]

Individual Differences in Premotor Brain Systems Underlie Behavioral Apathy — Individual Differences in Premotor Brain Systems Underlie Behavioral Apathy — Supplementary Data 

# Individual Differences in Premotor Brain Systems Underlie Behavioral Apathy

## Supplementary Data

Supplementary Data

- Supplementary Data - Docx file
