# Supplementary material for: Individual Differences in Premotor Brain Systems Underlie Behavioral Apathy
Source: Cereb Cortex. 2015 Nov 12;26(2):807–19. doi: 10.1093/cercor/bhv247 (PMC4712805; doi:10.1093/cercor/bhv247)
Supplement: Supplementary Data [file supp_bhv247_bhv247supp.docx]

**Supplementary Material**

**Methods**

*Questionnaires*

Self-reports of apathy traits were obtained using a modified, extended version of the original Lille apathy rating scale (LARS-e), which consists of a 51-item questionnaire assessing four domains of apathy: Action Initiation (AI), Intellectual Curiosity (IC), Emotional Responsiveness (ER) and Self-Awareness (SA). Participants can rate how much the different statements reflect their own behavior, habits or personality traits on a 1 to 5 Likert-scale, 1 corresponding to extremely apathetic and 5 extremely motivated. The Action AI subscale, which measures every-day productivity and initiative (two highly correlated subcomponents that were subsequently collapsed to compute AI scores), was of particular interest to us as an index of behavioral apathy (when scores are reverted, i.e. 5 – AI), which refers to diminished self-initiated actions, lack of effort and decrease in productivity (Marin and Wilkosz 2005).

*Task description*

In order to efficiently model choice behavior, participants also performed two blocks of the task outside the scanner. To maximize data sampling of incentive/effort combinations where choice varies the most (i.e. close to indifference points), we used an adaptive algorithm so that the trees presented on a given trial depended on participant’s previous choices. If a combination was refused on one trial, then on a subsequent trial, the incentive would increase, or the effort level would decrease (incentive and effort were adjusted alternatively).

In addition, we controlled for the number of effortful responses. If participants gave an effortful response on more than 50% of the previous trials, they would be presented with the screen: ‘No response required, get ready for next trial’ if they selected the YES option. This procedure allowed limiting fatigue effects, and controlling for the number of effortful responses. As a result of this procedure, individuals who responded ‘yes’ more often saw the ‘No response required’ message more often (supplementary Figure 4).

*Control for learning effect*

To ensure there was no change of reward sensitivity over time, we split our behavioral data into two halves, and performed a repeated measure 2x2 ANOVA to quantify the effect of the interaction time*reward-sensitivity. There was no significant effect of time on reward sensitivity (F=0.818, p = 0.373).

In addition, to rule out the possibility that learning may be related to behavioral apathy, we split our participants into two groups according to their AI (action initiation) scores on the LARSe questionnaire and performed a 2x2x2 ANOVA. There was no significant interaction of time*reward-sensitivity*Apathy group (p=0.235, F=1.470).

**References**

Marin RS, Wilkosz PA. 2005. Disorders of diminished motivation. J Head Trauma Rehabil. 20:377–388.

**Supplementary Figure 1**

**
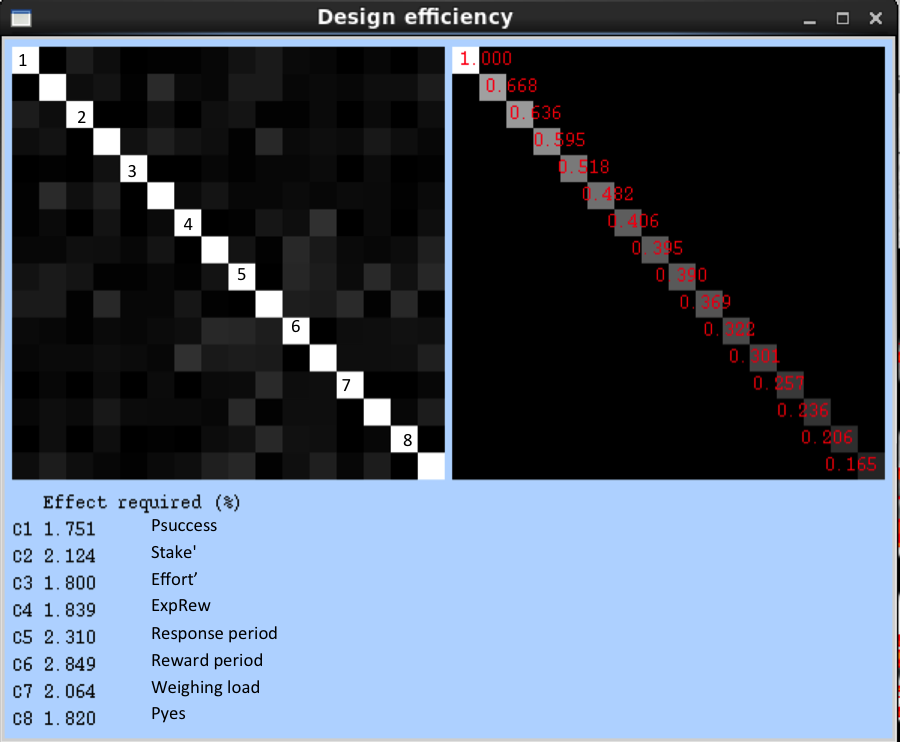
**

**Supplementary Figure 1: FMRI design efficiency**

Design efficiency report produced in FSL FEAT GUI: The top left matrix shows the absolute value of the normalised correlation of each EV with each EV (and their temporal derivative). Lighter shades of grey indicate higher level of correlation between EVs. The second matrix shows a similar thing after the design matrix has been run through SVD (singular value decomposition). All non-diagonal elements will be zero and the diagonal elements are given by the eigenvalues of the SVD, so that a poorly-conditioned design is obvious if any of the diagonal elements are black.

In the lower part of the window, for each requested contrast, that contrast's efficiency/estimability is shown. This is formulated as the strength of the signal required in order to detect a statistically significant result for this contrast. This Effect Required depends on the design matrix, the contrast values, the statistical significance level chosen, and the noise level in the data. The lower the effect required, the more easily estimable is a contrast, i.e. the more efficient is the design.

**Supplementary Figures**

**Supplementary Figure 1**

**
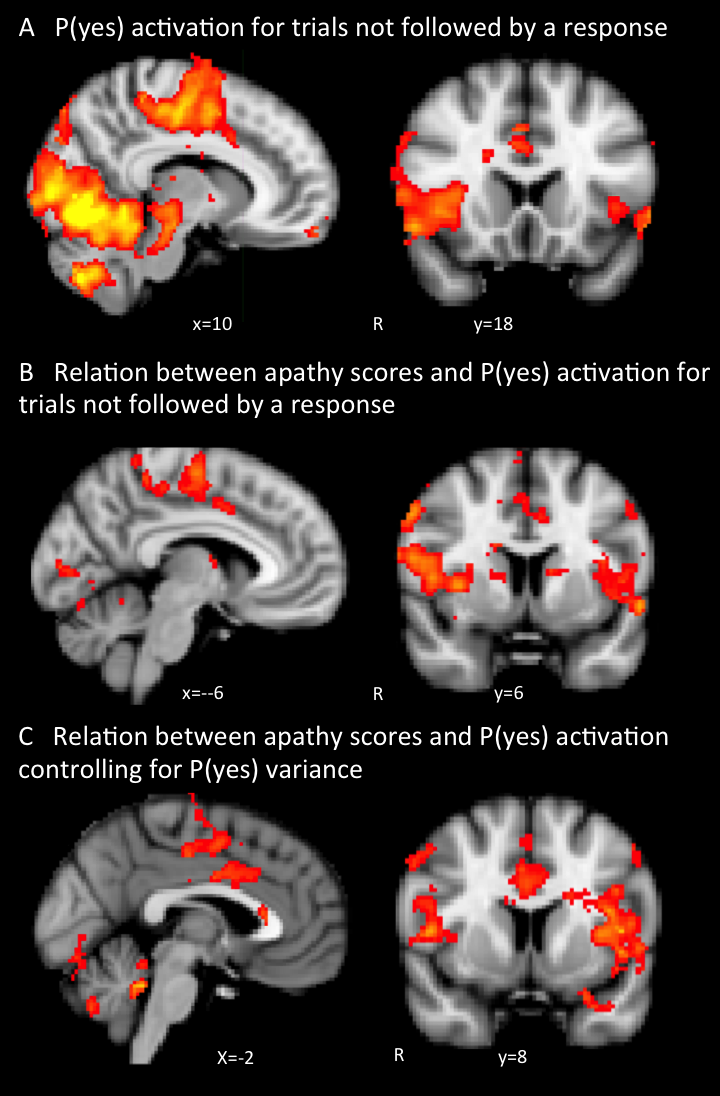
**

**Supplementary Figure 1: Control analyses for P(yes) BOLD signal interpretation**

BOLD signal increase with increase probability to engage in an effortful response (increase P(yes)), A) on trials that are not followed by an effortful motor response (response is no or trial aborted after accepting an offer), B) in relation with apathy traits on trials that are not followed by an effortful motor response and C) in relation with apathy traits while controlling for P(yes) regressor variance. Final statistical images were thresholded by using Gaussian Random Field based cluster inference with a height threshold of Z > 2.3 and a cluster significance threshold of P<0.05

**Supplementary Figure 2**

**
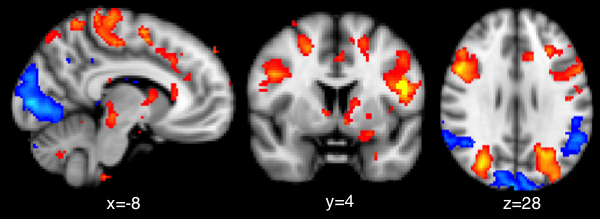
**

**Supplementary Figure 2: Functional connectivity with SMA**

Statistical map of regions showing significant positive (yellow-orange) and negative (blue) correlation with signal fluctuation within the SMA during the decision period on accepted offer trials. z threshold = 2.3 and cluster significance threshold p=0.05.

**Supplementary Figure 3**


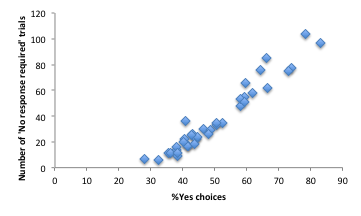


**Supplementary Figure 3**: Relation between %Yes and number of ‘No response required trials’ plotted for each participant.

**Supplementary Tables**

**Supplementary Table1:** Subject’s behavioral model parameters estimates and model likelihood (AIC)

| Subject | ß0 | ßStake | ßEffort | ßExpRew | ßPsucc | AIC |
| --- | --- | --- | --- | --- | --- | --- |
| 1 | -1.04 | -0.69 | 0.74 | -4.57 | -0.03 | 211.48 |
| 2 | -0.84 | -0.8 | 1 | -1.16 | -0.3 | 263.86 |
| 3 | 0.1 | -0.46 | 0.49 | -1.3 | -0.03 | 264.85 |
| 4 | -0.15 | 0.24 | -3.08 | -4.93 | 5.74 | 164.5 |
| 5 | -1.45 | 0.34 | 0.77 | -3.34 | -0.18 | 211.57 |
| 6 | -1.97 | -0.22 | 0.94 | -2.62 | 0.13 | 190.56 |
| 7 | 1.01 | -4.96 | 4.63 | -6.53 | -1.25 | 146.88 |
| 8 | 0.43 | 0.27 | 1.01 | -3.51 | -0.35 | 216 |
| 9 | -1.22 | -3.03 | 3.19 | -6.82 | 0.23 | 144.92 |
| 10 | 0.7 | -2.61 | 3.43 | -6.14 | 0.16 | 141.53 |
| 11 | -1.4 | -1.52 | 2.29 | -5.87 | 0.12 | 145.83 |
| 12 | -0.23 | -2.07 | 1.76 | -5.21 | 0.56 | 141.05 |
| 13 | -0.59 | 1.36 | -0.03 | -3.05 | -3.75 | 175.95 |
| 14 | 0.89 | -8.77 | 8.73 | -9.44 | -1.16 | 130.77 |
| 15 | -0.08 | -2.81 | 6.65 | -7.5 | -0.73 | 120.64 |
| 16 | -0.65 | -1.11 | 2.57 | -7.6 | -0.01 | 135.39 |
| 17 | -0.39 | -1.73 | 0.25 | -7.85 | 0.14 | 120.51 |
| 18 | 0.75 | -3.29 | 1.4 | -4.62 | -0.13 | 164.81 |
| 19 | -0.28 | -0.3 | 1.63 | -5.01 | -1.32 | 183.59 |
| 20 | 0.3 | -0.29 | 0.73 | -3.73 | -0.01 | 202.29 |
| 21 | -4.2 | -8.87 | 7.63 | -17.7 | -0.39 | 71.49 |
| 22 | 0.86 | -0.62 | 3.37 | -5.2 | -0.45 | 144.15 |
| 23 | -0.59 | -4.78 | 1.29 | -10.09 | -0.72 | 87.22 |
| 24 | 0.6 | -1.91 | 2.33 | -5.67 | -0.17 | 116.4 |
| 25 | 1.91 | -3.59 | 3.17 | -7.63 | -0.75 | 108.94 |
| 26 | 0.95 | -3.56 | 4.9 | -8.4 | 0.25 | 105.04 |
| 27 | -1.67 | -2.4 | 1.49 | -2.57 | -1.7 | 169.01 |
| 28 | -0.84 | -0.72 | 1.89 | -3.12 | 0.36 | 207.05 |
| 29 | 0.17 | -4.07 | 2.6 | -5.85 | -1 | 147.96 |
| 30 | 0.71 | -1.81 | 2.59 | -3.82 | 0.2 | 198.43 |
| 31 | 2.07 | 0.37 | 0.24 | -5.92 | 0.91 | 124.65 |
| 32 | 1.03 | -1.62 | 2.79 | -7.17 | 0.58 | 118.51 |
| 33 | 0.41 | 0.36 | 0.63 | -1.84 | 0.23 | 209.12 |
| 34 | -2.04 | -2.14 | 2.1 | -5.63 | -1.4 | 123.35 |
| 35 | -0.96 | 0.35 | 2.63 | -3.74 | -0.36 | 170.45 |
| 36 | -2.69 | -1.13 | 4.76 | -4.49 | 0.17 | 128.74 |
| 37 | -2.64 | -1.85 | 2.34 | -2.36 | -0.59 | 121.03 |

**Supplementary Table 2:** Behavioral results/correlation apathy

|  | Correlation coefficients with behav apathy scores |
| --- | --- |
|  |  |
| *Model* |  |
| ß0 | r=0.06, p=0.69 |
| ßStake | r=-0.293, p=0.08 |
| ßEffort | r=0.363, p=0.03* |
| ßExpRew | r=-0.193, p=0.26 |
| ßPsuccess | r=-0.08, p=0.65 |
| AIC | r=0.13, p=0.45 |
| *Other task measures* |  |
| Average Force exerted | r=-0.005, p=0.98 |
| %Yes choices | r=-0.11, p=0.53 |
| RT(yes) | r=-0.17, p=0.33 |
| RT(no) | r=-0.18, p=0.29 |

**Supplementary Table 3**: Local maxima for the fMRI group analysis for Stake-, Effort-, and Expected reward-related BOLD signal change

|  | MNI coordinates | | | z-stats |
| --- | --- | --- | --- | --- |
|  | x | y | z |  |
| **Stake** |  |  |  |  |
| Occipital pole | 0 | -90 | 2 | 4.14 |
| R IFG/ant Insula | 44 | 18 | 6 | 4.07 |
| R Frontal pole | 36 | 60 | -4 | 3.82 |
| L Postcentral gyrus | -56 | -12 | 48 | 3.74 |
| **Effort** |  |  |  |  |
| R mid Insula/putamen | 36 | -6 | 4 | 5.22 |
| R inferior temporal gyrus | 46 | -46 | -14 | 5.21 |
| L Precentral gyrus | -50 | -4 | 48 | 5.1 |
| L Putamen | -18 | 14 | 0 | 5.01 |
| R Accumbens/Caudate | 6 | 4 | 0 | 4.94 |
| R IFG | 42 | 32 | 16 | 4.9 |
| posterior cingulate gyrus | 6 | -40 | 20 | 4.36 |
| Cingulate motor area | 4 | -2 | 38 | 4.22 |
| R Accumbens | 14 | 18 | -10 | 4.22 |
| **Expected Reward** |  |  |  |  |
| L Postcentral gyrus | -58 | -10 | 24 | 3.69 |
| L Caudate | -10 | 2 | 4 | 3.45 |
| R Postcentral gyrus | 54 | -20 | 58 | 3.54 |
| R Caudate | 12 | 12 | 6 | 3.16 |
| L Putamen | -26 | -12 | 6 | 3.22 |
| L SMA | -10 | -18 | 50 | 3.52 |

**Supplementary Table 4:** Local maxima for the fMRI group analysis for Weighing load and Probability of accepting an offer, positively and negativelyrelated BOLD signal change

|  | MNI coordinates | | | z-stats |
| --- | --- | --- | --- | --- |
|  | x | y | z |  |
| **Weighing load** |  |  |  |  |
| *positive correlation* |  |  |  |  |
| R middle frontal gyrus | 42 | 30 | 36 | 4.73 |
| R Angular gyrus | 50 | -52 | 48 | 3.94 |
| L Superior parietal cortex | -52 | -60 | 50 | 3.65 |
| pSMA/dACC | 0 | 34 | 38 | 3.94 |
| *negative correlation* |  |  |  |  |
| vMPFC | -4 | 56 | -6 | 4.26 |
| Precuneus/PCC | -6 | -54 | 20 | 3.64 |
| **P(yes)** |  |  |  |  |
| *positive correlation* |  |  |  |  |
| rIFG/precentral gyrus | 52 | 10 | 8 | 6.79 |
| Motor cingulate area | -16 | -32 | 38 | 6.75 |
| L Lingual gyrus | -18 | -56 | -4 | 6.58 |
| R frontal pole | 24 | 54 | -12 | 6.46 |
| SMA | 4 | -4 | 52 | 6.18 |
| mid-cingulate gyrus | 6 | 8 | 36 | 6.07 |
| R mid Insula | 40 | 6 | 0 | 6.09 |
| R Pallidum | 18 | -4 | -4 | 5.74 |
| R Thalamus | 16 | -18 | 16 | 5.75 |
| R putamen | 28 | -12 | 6 | 5.33 |
| R ant Insula | 32 | 18 | 8 | 5.65 |
| *negative correlation* |  |  |  |  |
| Sugenual cingulate gyrus | -2 | 24 | -12 | 4.8 |

**Supplementary Table 5:** Local maxima of brain activation where BOLD signal is correlated with behavioral apathy traits.

|  | MNI coordinates | | | z-stats |
| --- | --- | --- | --- | --- |
|  | x | y | z |  |
| **Positive correlation Apathy scores/Effort-related signal change** | | | | |
| R Caudate | 14 | 24 | 0 | 3.34 |
| Cingulate motor area | 12 | -6 | 38 | 3.19 |
| mid Cingulate gyrus | 12 | 16 | 34 | 3.01 |
| R Lateral orbito-frontal cortex | 44 | 44 | 4 | 2.92 |
| R Accumbens | 12 | 20 | -4 | 2.67 |
| **Positive correlation Apathy scores/P(yes)-related signal change** | | | | |
| R precentral gyrus | 22 | -24 | 62 | 4.91 |
| L parietal operculum | -52 | -28 | 22 | 4.90 |
| R caudate | 16 | 8 | 12 | 4.82 |
| R post Insula | 40 | -10 | 12 | 4.78 |
| L post Insula | 42 | -2 | 0 | 4.35 |
| rczp | 2 | 6 | 30 | 4.14 |
| L ant Insula | -36 | 10 | 4 | 3.91 |
| SMA | 2 | -2 | 52 | 3.82 |
| R Accumbens | 12 | 10 | -8 | 3.54 |

**Supplementary Table 6:** Peaks of significant positive and negative correlation with signal fluctuation within the SMA during the decision period on accepted offer trials (PPI analysis).

|  | MNI coordinates | | | z-stats |
| --- | --- | --- | --- | --- |
|  | x | y | z |  |
| *positive correlation* |  |  |  |  |
| R Superior frontal gyrus | 22 | 4 | 50 | 4.03 |
| L Superior frontal gyrus | -22 | -2 | 52 | 3.32 |
| L Superior parietal cortex | -36 | -50 | 54 | 3.98 |
| R Superior parietal cortex | 30 | -46 | 54 | 3.94 |
| SMA | -10 | 2 | 54 | 3.72 |
| L Inferior frontal gyrus | -46 | 4 | 24 | 3.61 |
| Paracingulate gyrus | -10 | 24 | 38 | 3.56 |
| Precentral gyrus | -2 | -24 | 54 | 4.26 |
| L subthalamic nucleus | -8 | -24 | -6 | 3.21 |
| R subthalamic nucleus | 6 | -24 | -8 | 3.34 |
| *L caudate* | -6 | 6 | 6 | 3.36 |
| *Negative correlation* |  |  |  |  |
| Lingual gyrus | 6 | -74 | 0 | 4.79 |
| R angular gyrus | 42 | -52 | 26 | 4.03 |
| L angular gyrus | -52 | -60 | 30 | 3.34 |
| Precuneus/Posterior cingulate cortex | 10 | -48 | 38 | 3.49 |
